# Social influence matters: We follow pandemic guidelines most when our close circle does

**DOI:** 10.1111/bjop.12491

**Published:** 2021-01-20

**Authors:** Bahar Tunçgenç, Marwa El Zein, Justin Sulik, Martha Newson, Yi Zhao, Guillaume Dezecache, Ophelia Deroy

**Affiliations:** ^1^ School of Psychology University of Nottingham UK; ^2^ Institute of Cognitive & Evolutionary Anthropology University of Oxford UK; ^3^ Institute of Cognitive Neuroscience University College London UK; ^4^ Adaptive Rationality Center Max‐Planck Institute for Human Development Berlin Germany; ^5^ Cognition, Values and Behaviour Lab Ludwig Maximilian University Munich Germany; ^6^ School of Anthropology and Conservation University of Kent UK; ^7^ School of Medicine Indiana University Bloomington Indiana USA; ^8^ CNRS, LAPSCO Université Clermont Auvergne Clermont‐Ferrand France; ^9^ Munich Center for Neuroscience Ludwig Maximilian University Munich Germany; ^10^ School of Advanced Study Institute of Philosophy University of London UK

**Keywords:** COVID‐19 pandemic, norm change, public health behaviour, social closeness, social distancing

## Abstract

Why do we adopt new rules, such as social distancing? Although human sciences research stresses the key role of social influence in behaviour change, most COVID‐19 campaigns emphasize the disease’s medical threat. In a global data set (*n* = 6,675), we investigated how social influences predict people’s adherence to distancing rules during the pandemic. Bayesian regression analyses controlling for stringency of local measures showed that people distanced most when they thought their close social circle did. Such social influence mattered more than people thinking distancing was the right thing to do. People’s adherence also aligned with their fellow citizens, but only if they felt deeply bonded with their country. Self‐vulnerability to the disease predicted distancing more for people with larger social circles. Collective efficacy and collectivism also significantly predicted distancing. To achieve behavioural change during crises, policymakers must emphasize shared values and harness the social influence of close friends and family.

## Background

To control the spread of COVID‐19, public adherence to the rules is critical. Campaigns promoting social distancing and other measures have aimed to persuade individuals that the threat is serious and that adherence to these measures will protect them from the disease. Yet, decades of human sciences research shows that a key driver of behaviour change is social influence from others: Humans are social cooperators, who construe behaviour change as a collective problem (Dezecache, Frith, & Deroy, 2020) and align more closely with those they are closely bonded to (Haun & Over, 2015).

We collected data from 115 countries (*n* = 6,675) to investigate whether social influence is associated with adherence to COVID‐19 guidelines. This study is unique in its consideration of both personal and social factors influencing people’s adherence to the social distancing rules. The few existing peer‐reviewed studies on social predictors of COVID‐19 adherence have found that people engage in more preventive measures when they have higher social responsibility and trust (Oosterhoff, Palmer, Wilson, & Shook, 2020) and when they consider adherence to the rules as a norm endorsed by others (Borgonovi & Andrieu, 2020; Lin et al., 2020; Nakayachi, Ozaki, Shibata, & Yokoi, 2020). While these studies point to the important role of social predictors in adherence to COVID‐19 measures, they have been limited due to their relatively narrow geographical and cultural scope. Besides, to our knowledge, no research has examined how different aspects of social norms predict adherence, taking into account degree of closeness to others (e.g., close social circle vs. fellow citizens). Yet, theory‐driven papers from diverse disciplines have consistently called for researchers and policymakers to consider the complex social influences from our close social circles and bonded communities (Andrews, Foulkes, & Blakemore, 2020; Bavel et al., 2020; Bonell et al., 2020; Jetten, Reicher, Haslam, & Cruwys, 2020; Prentice & Paluck, 2020; Prosser, Judge, Bolderdijk, Blackwood, & Kurz, 2020).

Addressing this theoretical gap with a rich data set, this paper examines how social influence affects adherence at three scales: one’s close circle, one’s country, and the entire world. Specifically, we compared people’s *own* adherence and approval of the rules to how much they believed *others* around them adhered and approved. We posit that across the three social scales, the degree of closeness with others will determine how much perceived adherence and approval of others impacts on one’s own adherence. We argue that while feelings of vulnerability to the disease would influence people’s adherence to the rules, in this context of social norm change, the strongest predictor of adherence would be the perceived adherence of one’s close circle.

Examinations of large, cross‐cultural databases reveal a distinct pattern observed in humans’ bond formation (Hill & Dunbar, 2003). On average, people tend to have about five others in their close circle, whom they turn to for advice or comfort during major life challenges (Hill & Dunbar, 2003). Extending beyond this primary social circle is larger circles of decreasing closeness, such as colleagues or fellow citizens (Hill & Dunbar, 2003). When projected on to larger groups (e.g., one’s country), these relational bonds can create a potent form of social bonding among virtual strangers (Swann, Gómez, Seyle, Morales, & Huici, 2009; Swann, Jetten, Gómez, Whitehouse, & Bastian, 2012). Such fusion of personal and group identities is often observed in times of extreme hardship (Whitehouse et al., 2017). Given the global nature of the pandemic, our survey captures the distinctive influences of three social scales (i.e., close circle, country, world) on people’s adherence to the emerging social norms around distancing.

Endorsement of public health behaviours during the pandemic is marked predominantly by social norm change, as COVID‐19 distancing rules diverge from the widely endorsed ways of social interactions and behaviour. The literature on social norms points to a distinction between descriptive norms and prescriptive, or injunctive, norms (Bicchieri, 2016; Cialdini & Goldstein, 2004). Descriptive norms refer to those social norms to which others adhere in practice. In contrast, prescriptive norms refer to those social norms which others approve in their discourse. Laboratory and field research suggest that when it comes to enacting behavioural change, actions speak louder than words: We are influenced by others if we think they also adhere to the rules, rather than simply approve of them (Bicchieri, 2016; Bicchieri & Xiao, 2009; Cialdini & Goldstein, 2004). Thus, our first hypothesis (Hypothesis 1) is that perceived adherence of others (i.e., descriptive norms) is a stronger predictor of self‐adherence than perceived approval of others (i.e., prescriptive norms).

Forming close emotional bonds with other group members is a strong determinant of how people identify themselves as belonging to one group over another (Tajfel & Turner, 1979; Whitehouse & Lanman, 2014). Group identities encourage people to like, trust, and cooperate more with members of their own group as compared to outsiders (Raafat, Chater, & Frith, 2009; Shamay‐Tsoory, Saporta, Marton‐Alper, & Gvirts, 2019) – even minimal groups, based on arbitrary categories can induce ‘in‐group’ biases (Goette, Huffman, & Meier, 2012; Tajfel, 1970). To learn about their group’s social norms and conventions, people observe and imitate other group members (Legare & Nielsen, 2015). Imitation and social bonds are intricately linked: Closeness breeds imitation, and imitation breeds closeness (Chartrand & Lakin, 2011; Haun & Over, 2015). When urgent behavioural change is needed, such as during the present pandemic, personal motives may be insufficient; instead, adoption of new norms may depend more strongly on the influence of close (Andrews et al., 2020; Bicchieri, 2016

; Goldstein, Cialdini, & Griskevicius, 2008). Therefore, our second hypothesis is that self‐adherence to COVID‐19 rules depends more on perceived adherence of one’s close circle than that of the outer social scales of country and world, or one’s own approval of the distancing rules (Hypothesis 2). Further, we hypothesize that perceived adherence of fellow citizens influences self‐adherence only when people are strongly fused with their country (Hypothesis 3).

During the pandemic, the material threat of contracting the disease is likely to influence people’s adherence to distancing measures. Reports from many countries have shown elevated levels of fear and anxiety following the COVID‐19 outbreak (de Pedraza, Guzi, & Tijdens, 2020). Notably, fear of COVID‐19 stems not only from perceiving one’s self as vulnerable to the disease, but also from perceiving loved ones as vulnerable (Mertens, Gerritsen, Duijndam, Salemink, & Engelhard, 2020). Therefore, we hypothesize that perceived vulnerability of close ones positively predicts self‐adherence to distancing in addition to perceived self‐vulnerability to the disease (Hypothesis 4).

Equally, fear can trigger social contact seeking and adaptive responses to threatening situations (El Zein, Wyart, & Grèzes, 2015; Harper, Satchell, Fido, & Latzman, 2020). When fear is coupled with social support (Gallagher, Luttik, & Jaarsma, 2011; Tang, Brown, Funnell, & Anderson, 2008) and belief in collective responsibility and efficacy (Witte & Allen, 2000), people are more likely to engage in constructive actions. Thus, in a fear‐inducing context such as the COVID‐19 pandemic, those who feel vulnerable to the disease may adhere to distancing rules more if they have stronger social support. Our final hypothesis is that the effects of vulnerability of self and others on self‐adherence are stronger for people with larger close circles (Hypothesis 5).

**Figure 1:**
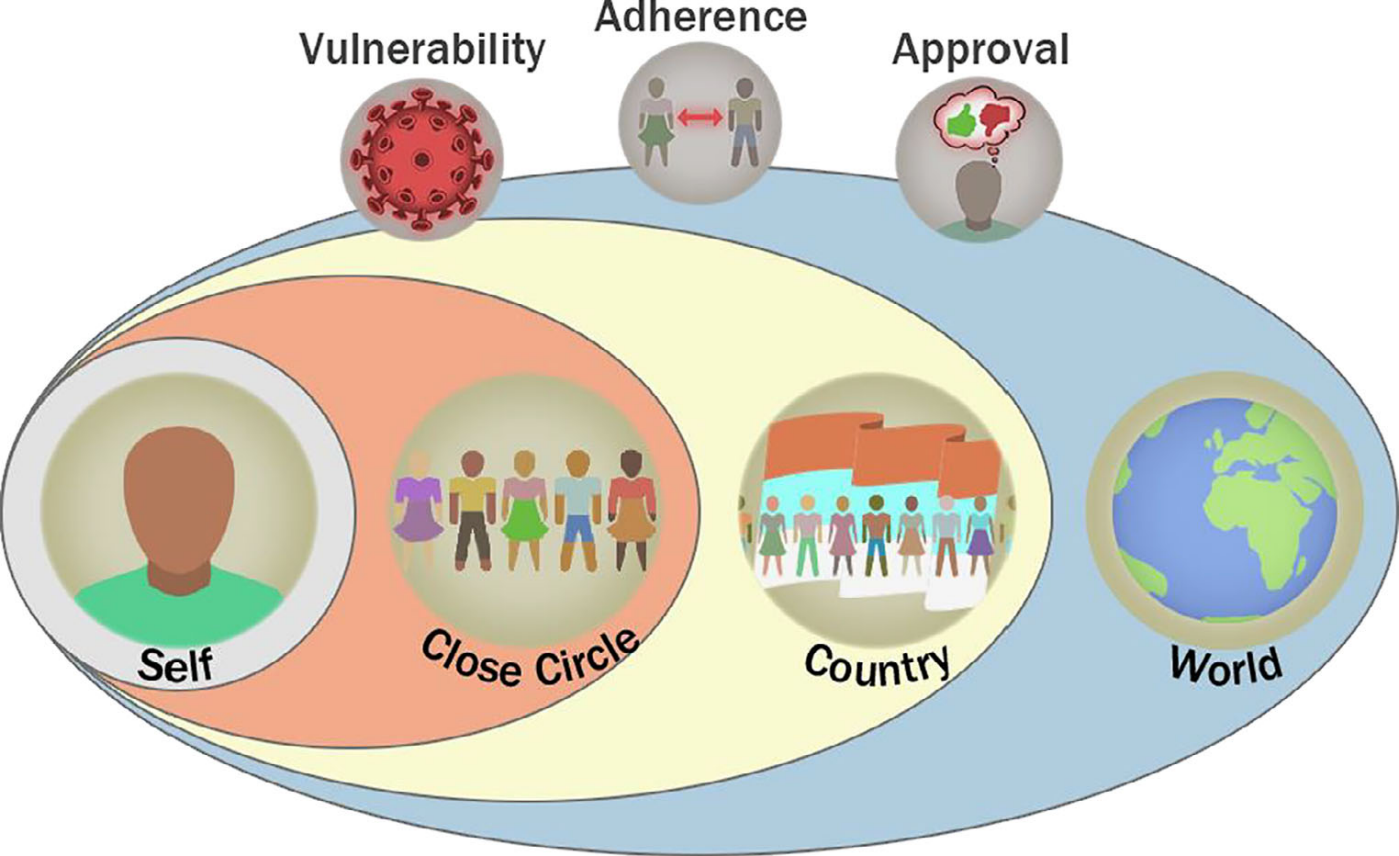
Proposed theoretical framework. The effects of perceived vulnerability to the disease, adherence to distancing rules, and approval of distancing rules (top row) operate on three social scales to predict self‐adherence: close circle, country, and world. In our framework, social influence, especially from our closest circle, outweighs our individual motives to adhere to distancing. How much we think others are also adhering to the rules influences our own behaviour more strongly than how much we think others are approving of the rules. The influence of the wider social scales (i.e., country and world) depends on how closely bonded one is to these groups. Social bonds also interact with feelings of vulnerability to the disease: Perceiving loved ones as vulnerable motivates us to adhere to the rules beyond our perceptions of self‐vulnerability to the disease. Finally, self‐vulnerability is likely to be more strongly linked with adherence when we receive more social support.

These five pre‐registered hypotheses https://osf.io/ke5yn/ form the basis of our framework (see Figure 1). In addition to these, we conducted an exploratory analysis examining a range of social orientation variables. Previous research suggests that people are more likely to participate in collective action when they believe that the responsibility lies with the collective, rather than with individuals, and that the efficacy of the collective’s actions is high (Zomeren & Iyer, 2009). In the context of COVID‐19, greater social responsibility and trust have been associated with less hoarding behaviour (Oosterhoff & Palmer, 2020). Other recent studies have also shown that having more collectivistic values and empathy can enhance people’s engagement with COVID‐19 containment measures (Miguel, Machado, Pianowski, de Carvalho, & F., 2021; Pedersen & Favero, 2020). To investigate the contributions of these factors, we included four additional variables in our analyses: (1) collective responsibility, (2) collective efficacy, (3) vertical collectivism, which defines willingness to sacrifice one’s self‐interests for one’s group (Singelis, Triandis, Bhawuk, & Gelfand, 1995), and (4) empathy quotient, which defines one’s ability to understand and align with others’ emotional states (Wakabayashi et al., 2006).

## Methods

### Ethics

The study was approved by the ethics committee of the University of Nottingham. Participants provided written informed consent prior to participation in line with the General Data Protection Regulation (GDPR). All participants were assigned an anonymous ID.

### Participants

In total, 6,675 people completed our survey. Participants could opt out of the questions that did not apply in their specific circumstances (i.e., if they had no one in their close circle: *n* = 1,199) or that involved personal information (i.e., fusion with country: *n* = 213; country of residence: *n* = 41). Thus, for each section of the results, we report the number of participants with complete responses for those specific variables (Results Sections 1 and 3: *n* = 5,335, Section 2: *n* = 6,634). Full demographics of our data sets can be found in Table S2. We repeated the Section 2 analysis on the reduced data set, which revealed the same findings (see Table S3).

On the survey landing page, participants chose which language they wished to take the survey in (options: Arabic, Bangla, German, English, Spanish, French, Hindi, Italian, Persian, Swedish, Turkish, Mandarin). This range of languages aimed at recruiting people from diverse cultural backgrounds, driven partly by the researchers' expertise and backgrounds.

#### Sampling strategy

Participants were recruited via announcements on social media, student mailing lists at the University of Nottingham, University of Oxford and Ludwig Maximilian University of Munich, the participant pool of RISC (France), press releases by the Universities of Nottingham and Oxford, and blog posts published in the UK, Germany, and Turkey. To obtain as widely and globally representative a sample as possible, we had a stopping rule of accepting responses within 5 weeks of a language becoming available, with the first language (English) published on 9 April 2020 and the last one (Hindi) on 29 April 2020.

### Materials and procedure

A demo version of the entire survey is available for viewing at the project’s OSF page at https://osf.io/ke5yn/. Full survey details can be found in SM Methods; below, we explain the measures used in this study (see Table 1). Not all measures in the survey are analysed for this study; the remaining measures will be analysed for future studies and available open access on the study’s OSF page.

**Table 1:** The variables reported in this study and descriptions of how they were measured

Variable name	Description/sample item and scoring
Close circle size	Number of people the participant would turn to for advice or comfort among the ones the participant indicated having voluntary contact within the past week
Adherence (sub‐categories: self, close circle, country, world)	‘I have been following this general advice where I live’ ‘Most people in my close circle/my country/the world have been following this general advice where they live/’ 100‐point scale from 0 = ‘not been following this advice at all’ through 50 = ‘Been following the advice exactly’ to 100 = ‘Been doing more than what is advised’
Approval (sub‐categories: self, close circle, country, world)	‘I think that it is wrong not to follow this general advice’ ‘Most people in my close circle/my country/the world think that it is wrong not to follow this general advice’ 100‐point scale from 0 = ‘Not following the advice is completely ok’ to 100 = ‘Not following the advice is completely wrong’
Vulnerability (sub‐categories: self, others)	100‐point scale from 0 = ‘Not vulnerable at all’ through 50 = ‘As vulnerable as an average person’ to 100 = ‘Extremely vulnerable’
Collective responsibility	‘At times like this, it is essential that people work in solidarity to look after each other’ ‘Every individual is responsible for themselves if they want to avoid the adverse effects of the disease’ (reverse coded) 0 = ‘Completely disagree’ to 100 = ‘Completely agree’
Collective efficacy	‘Things are improving due to the collective efforts made where I live’ ‘I believe my actions are having a positive impact’ (reverse coded) 0 = ‘Completely disagree’ to 100 = ‘Completely agree’
Fusion with country	5‐point pictorial scale depicting self and country in increasing degree of overlap; scored as 1 if self and country completely overlap, 0 otherwise
Vertical collectivism	‘I usually sacrifice my self‐interest for the benefit of my group’ 1 = ‘Never’ to 10 = ‘Always’
Empathy quotient	‘I tend to get emotionally involved with a friend's problems’ 1 = ‘Strongly disagree’ to 4 = ‘Strongly agree’

#### Close circle

Following prior work (Dunbar & Spoors, 1995), we obtained the size of participants’ close circle by first asking them to enter the first names of people they had voluntarily had a conversation with in the past week, and then, asking them which of these contacts they would turn to for comfort or advice with a major personal problem (see Figure S1A). The names entered were not retained in our data set and were only used to extract the number of people in each category. Participants could skip these questions if they had no such interaction. The measure close circle used in this study is the number of people that participants indicated they would turn to for advice or comfort.

#### Social norm change

Participants were first reminded that the general advice for COVID‐19 was to keep physical distance from others. As our measure of adherence to the rules, we asked participants to indicate on slider scales how much they were following this general advice, with options ranging from ‘1 = have not been following the advice at all’ through ‘50 = have been following the advice exactly’ to ‘100 = have been doing more than what is advised’. Confirming construct validity, this item was significantly correlated, *r*(5474) = −.21, *p* < .0001 with another item measuring distancing behaviour, in which participants rated how much they have been going out in the past week on a continuous slider scale ranging from ‘1 = much less than usual’ through ‘50 = about the same as usual’ to ‘100 = much more than usual’.

As our measure of approval of the rules, we asked how much participants thought it would be wrong not to adhere to the general advice in the past week, with options ranging from ‘1 = not following the advice is completely ok’ to ‘100 = not following the advice is completely wrong’.

The adherence and approval questions were then repeated for the three social scales in a randomized order: participant’s close circle (with a reminder of the names they provided), the people in the participant’s country, and people around the world (see Table 1 for exact wording of the items).

Our social norm change questions were contextually sensitive as the participants were asked to answer depending on how the advice was *currently* *applied* *where* *they* *lived*. Our models further contain a metric of local stringency measures (Hale et al., 2020) to account for contextual variability.

#### Vulnerability

Participants were asked ‘In your opinion, how vulnerable are the following people to the coronavirus disease?’ and were given the categories: ‘Myself’, ‘Someone I care about in my household’ and ‘Someone I care about outside of my household’. These three items were answered on continuous slider scales, with the extreme ends labelled: ‘Not vulnerable at all’ and ‘Extremely vulnerable’. Two measures from these questions are used in this study: vulnerability_self_ and vulnerability_others_. Given that in‐household vulnerability levels would be strongly correlated with self‐vulnerability, we orthogonalized the in‐household vulnerability ratings by regressing them on the self‐vulnerability ratings and taking the residuals. Then, we averaged these scaled residuals with rescaled outside‐household ratings to yield an overall rating of participants’ perception of others' vulnerability to the disease, which made up the score for the vulnerability_others_ variable.

#### Social orientation

Participants answered 4 purpose‐made items using 100‐point continuous slider scales to indicate how much they agreed with statements describing the collective responsibility of their country and the collective efficacy of the actions being taken in response to the COVID‐19 pandemic (see Table 1 for items).

Participants used Likert‐type scales to respond to two previously established scales: the 8‐item Vertical Collectivism sub‐scale (Singelis et al., 1995), measuring how much people are willing to sacrifice self‐interests for others, and the 15‐item shortened Empathy Quotient (Wakabayashi et al., 2006), measuring people’s ability to understand others’ emotions and mental states (see Table S1 for the questionnaire items). All non‐English versions of the vertical collectivism scale and the Bangla version of the EQ scale were translated and back‐translated by native speakers’ proficient in English before use.

#### Fusion with the country

Participants rated their degree of fusion with their country on a 5‐point pictorial scale (Figure S1B) showing two circles representing self and country in gradually increasing degrees of overlap. In line with previous research (Swann et al., 2009), participants were considered ‘fused’ if they selected the total overlap option, and ‘not fused’ otherwise.

#### Demographics

Participants provided their age, gender (‘man’, ‘woman’, ‘non‐binary’, ‘prefer not to say’), highest completed education (‘No schooling completed’, ‘Primary education (age: 5–10)’, ‘Secondary education (age: 11–17)’, ‘University undergraduate degree/professional equivalent’, ‘Postgraduate degree’), current student and employment status, and whether they were studying/working from home (‘yes’, ‘sometimes’, ‘no’). Participants were also asked of their country of residence at the time of answering, which was used to obtain the stringency of lockdown measures in that country using the OxCGRT database (Hale et al., 2020).

### Statistical analysis

All analyses were conducted using open‐source R software version 1.3.959 (R Core Team, 2019) with package *brms* version 2.13.3 (Bürkner, 2018). The variables were scaled to *SD* = 1 and centred.

To control for the stringency of lockdown measures in our participants’ country of residence (and state, in the case of USA), we obtained the average stringency index score (Hale et al., 2020 of the 15 days preceding the day participants filled out the survey.

We conducted mixed‐effects Bayesian linear regressions with weakly informative priors for the model betas (β~*N*(0, 1)) to test our hypotheses. Details on the model priors, random effects structures, distribution plots, and other model fit measures can be found in the SM. All analyses included the participant’s country of residence as a random effect and the covariates of participant age, gender, education level (four levels), time spent outside of the home (three levels), and country’s/state’s stringency of lockdown measures. The R script used for analysis can be found on the study’s OSF page.

## Results

### The role of social adherence, social approval, and personal approval in self‐adherence

We examined our hypotheses regarding the role of social influence on adherence in two models: the social adherence model and the social approval model. These models assessed whether self‐adherence to distancing (adherence_self_) could be predicted by the perceived adherence or perceived approval of others at three social scales: close circle (recent contacts whom participants said they would turn to in hardship), country (fellow citizens), and world (humankind). Both models included people’s own approval of the distancing rules as a predictor, demographic variables and stringency of local COVID‐19 measures as covariates, and participants’ country of residence as a random effect. Participants who indicated not having contacted anyone in their close circle in the past week (*n* = 1,199) had missing data for the adherence and approval variables of close circle and were therefore excluded from these analyses. Of note, in line with the pre‐pandemic literature (Hill & Dunbar, 2003), participants in our sample had a median close circle size of 4 people.

**Figure 2:**
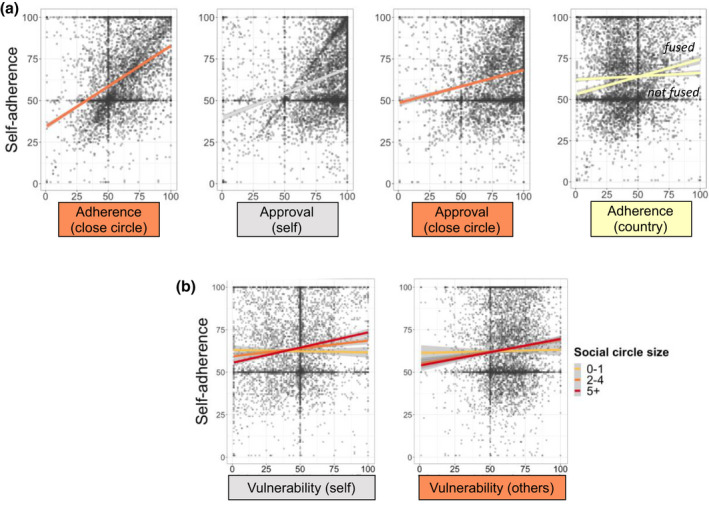
The roles of social adherence, social approval, self‐approval, and perceived vulnerability in people’s adherence to distancing rules. (A). How much one’s close circle adheres to distancing is a better predictor of self‐adherence than one’s own approval, which is a better predictor than the approval of the close circle. How much fellow citizens in one’s country adhere to distancing predicts self‐adherence only when one feels fused with their country. (B) Perceived vulnerability of self and others to the disease predicts self‐adherence more for people with larger close circles.

Detailed results of both the social adherence and the social approval models can be found in Table 2. The most influential predictors of self‐adherence are depicted in Figure 2A. For predictors with credible intervals abutting or including zero, we conducted additional hypothesis tests (using function *hypothesis* in R package *brms*) to provide Bayes Factors (BFs) quantifying the evidence supporting our claims. For directional hypotheses (e.g., ‘x has a positive effect on y’) BF_10_ = 3 means that a positive effect was 3 times more probable than a negative effect. For point hypotheses (e.g., ‘*x* had no effect on *y’*), BF_01_ = 3 means a beta of 0 was 3 times more likely given the data than it was before the model considered any data. As an informal rule of thumb, the frequentist convention of *p* < .05 can be mapped loosely onto BF ≈ 3, with higher BFs implying more confidence in the claims.

**Table 2:** Results of the Bayesian linear regressions predicting participant adherence to distancing (adherence_self_)

Predictors	β	*SE*	95% Credible intervals
Social adherence model: *R* ^2^ = 30.29% [28.50, 32.04]			
Approval_self_	.31	0.03	[0.25, 0.38]
Adherence_close circle_	.38	0.03	[0.33, 0.44]
Adherence_country_	−.01	0.02	[−0.04, 0.03]
Fusion	−.06	0.03	[−0.12, 0.00]
Adherence_country_ × fusion	.06	0.03	[0.00, 0.11]
Adherence_world_	.04	0.01	[0.02, 0.07]
Social approval model: *R* ^2^ = 16.19% [14.49, 17.93]			
Approval_self_	.36	0.04	[0.26, 0.43]
Approval_close circle_	.05	0.02	[0.01, 0.09]
Approval_country_	−.05	0.02	[−0.08, −0.02]
Fusion	−.05	0.03	[−0.12, 0.02]
Approval_country_ × fusion	.04	0.03	[−0.03, 0.10]
Approval_world_	−.03	0.02	[−0.06, 0.00]
Vulnerability model: *R* ^2^ = 5.99% [4.89, 7.12]			
Vulnerability_self_	.12	0.01	[0.09, 0.14]
Vulnerability_self_ × close circle	.06	0.01	[0.03, 0.08]
Vulnerability_others_	.10	0.01	[0.08, 0.13]
Vulnerability_others_ × close circle	.01	0.01	[−0.01, 0.04]
Exploratory model: *R* ^2^ = 32.64% [30.89, 34.37]			
Approval_self_	.27	0.03	[0.21, 0.33]
Adherence_close circle_	.38	0.03	[0.33, 0.44]
Adherence_country_	.02	0.02	[−0.02, 0.05]
Fusion	−.06	0.03	[−0.12, −0.01]
Adherence_country_ × fusion	.06	0.03	[0.00, 0.12]
Adherence_world_	.04	0.01	[0.01, 0.07]
Vulnerability_self_	.07	0.01	[0.04, 0.09]
Close circle	−.01	0.01	[−0.03, 0.02]
Vulnerability_self_ × close circle	.03	0.01	[0.00, 0.05]
Vulnerability_others_	.04	0.01	[0.02, 0.07]
Vulnerability_others_ × close circle	.00	0.01	[−0.03, 0.03]
Collective responsibility	−.01	0.01	[−0.03, 0.02]
Collective efficacy	.12	0.02	[0.07, 0.17]
Collectivism	.03	0.01	[0.00, 0.06]
Empathy	.01	0.01	[−0.01, 0.04]

The findings from the social adherence model support our hypothesis that the perceived adherence of one’s close circle had the strongest impact (β = .38 [0.33, 0.44]), followed by one’s own approval of the rules (β = .31 [0.25, 0.38]), with the adherence of people around the world (β = .04 [0.02, 0.07]) having a weaker effect. As hypothesized, we also found that perceived adherence of fellow citizens influenced self‐adherence only for people closely bonded, or fused, with their country (interaction β = .06 [0, 0.11]). The BF analysis in the social adherence model showed very strong evidence for there being no main effect of adherence_country_ (BF_01_ = 58), strong evidence for a negative effect of fusion with the country (BF_10_ = 57), and strong evidence for a positive interaction between adherence_country_ and fusion (BF_10_ = 32). Further, adherence_close circle_ had a greater effect than approval_self_ (BF_10_ = 10).

The findings from the social approval model showed that, when the impact of approval of the rules is considered on adherence, personal approval was the strongest predictor (β = .36 [0.29, 0.43]). As hypothesized, perceived approval of one’s close circle had a positive effect on self‐adherence (β = .05 [0.01, 0.09]), unlike the negative effects of perceived approval of fellow citizens (β = −.05 [−0.08, −0.02]) or people in the world (β = −.03 [−0.06, 0.00]). Examination of the BF for predictors with CIs near or including zero in the social approval model revealed strong evidence for a negative the effect of approval_world_ (BF_10_ = 24), and moderate evidence for there being no effect of fusion with the country (BF_01_ = 10), and for no interaction between approval_country_ and fusion (BF_01_ = 16).

Using approximate leave‐one‐out cross validation (Vehtari, Gelman, & Gabry, 2017), we compared the social adherence model to the social approval model. In line with our hypothesis, the results revealed that the social adherence model (*R^2^* = 30.29% [28.50, 32.04]) was a better fit than the social approval model (*R^2^* = 16.19% [14.49, 17.93]), with an estimated difference in expected log‐predictive density of 486.6 (*SE* = 37.1).

Together, these findings support our hypotheses by showing that perceived adherence of others was a better predictor of self‐adherence than perceived approval of others. Among different social scales (i.e., close circle, country, world), the perceived adherence and approval of one’s close circle were the most important determinants of self‐adherence, with the close circle’s adherence being even more important than one’s own approval of the distancing rules. Finally, perceived adherence of fellow citizens impacted self‐adherence only for people fused with their country.

### The role of perceived vulnerability of self and close others in self‐adherence

Perception of vulnerability was assessed with participant ratings of how vulnerable they considered themselves (vulnerability_self_) and others close to them (vulnerability_others_) to contracting the disease, on a continuous slider scale. We conducted a Bayesian linear regression with vulnerability_self_, vulnerability_others_ and their interactions with close circle size as the predictors, and adherence_self_ as the outcome variable. The results of this regression are reported in Table 2, and the interaction effects are depicted in Figure 2B. Supporting our hypothesis, we found that perceived vulnerability of close others (β = .10 [0.08, 0.13]) predicted adherence in addition to the effect of perceived self‐vulnerability (β = .12 [0.09, 0.14]). Moreover, in support of our hypothesis, perceived vulnerability of self was more strongly associated with self‐adherence for people with a larger close circle (β = .06 [0.03, 0.08]). Further hypothesis testing indicated strong evidence that there was no interaction between social circle size and vulnerability_others_ (BF_01_ = 45), suggesting that the association between perceived vulnerability of others and self‐adherence did not depend on the size of people’s close circle.

### Exploratory model comparing all contributors of self‐adherence

To examine how additional factors tap into one’s way of relating to the social environment predicted distancing, we extended the social adherence and vulnerability models with four additional variables. The adherence and approval variables within each social scale (i.e., close circle, country, and world) were strongly correlated with each other, close circle: *r*(5,364) = .39; country: *r*(5,364) = .45; world: *r*(5,364) = .36, all *p*s < .0001. Since our previous findings had shown the social adherence model to be a better fit for self‐adherence than the social approval model, in the current regression, adherence_self_ was regressed on: approval_self_, adherence_close circle_, adherence_country_ (and its interaction with fusion to country), adherence_world_, vulnerability_self_, vulnerability_others_ (the latter two in interaction with close circle size), collective responsibility, collective efficacy, collectivism, and empathy. As before, the model included participant age, gender, education level, time spent at home, and stringency of lockdown measures in country as covariates.

**Figure 3:**
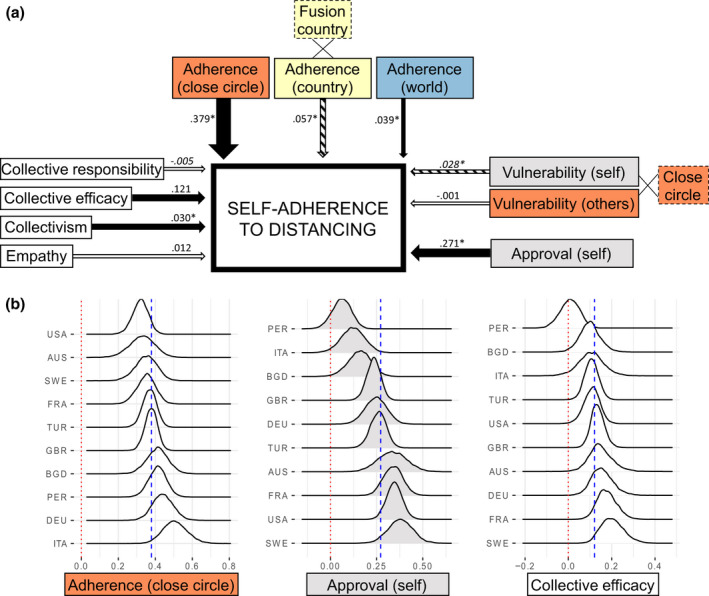
Exploratory model examining predictors of self‐adherence to distancing. (A) Dotted borders indicate variables that were entered as an interaction term in the model (i.e., fusion with country, close circle size). Black arrows (direct effect) and stripy arrows (interaction effect) indicate CIs excluding zero, white arrows indicate CIs including 0, and arrow width indicates the strength of the effect. (B) Density plots showing the top three predictors of the exploratory model (i.e., perceived adherence of close circle, self‐approval of the rules, and collective efficacy) in the top 10 countries with largest sample sizes. Blue dotted lines indicate the model estimate for the entire global dataset. AUS = Australia, BGD = Bangladesh, DEU = Germany, FRA = France, GBR = Great Britain, ITA = Italy, PER = Peru, SWE = Sweden, TUR = Turkey, USA = United States of America.

The results of this exploratory model are presented in Table 2. Detailed results of this model can be found in Figure 3A. The exploratory model revealed (*R* ^2^ = 32.64% [30.89, 34.37]) that the top 3 predictors of self‐adherence to distancing were as follows: adherence_close circle_ (β = .38 [0.33, 0.44]), followed by approval_self_ (β = .27 [0.21, 0.33]) and collective efficacy (β = .12 [0.07, 0.17]). The cross‐country variation in how these top 3 predictors predicted self‐adherence (for the top 10 countries with largest sample sizes, *n* ranging from 103 to 1,829) are illustrated in Figure 3B. This visualization highlights two important points: The relationships are in a consistent direction across countries, yet there is substantial variation between countries.

Overall, our exploratory analysis confirms that close circle’s perceived adherence has a pivotal role in determining self‐adherence. It also shows how own approval of the rules and belief in the collective efficacy of actions taken against the pandemic importantly predict self‐adherence.

## Discussion

This paper examined how social influence at different scales of closeness (i.e., close circle, country and world) impacted adherence to a central COVID‐19 strategy, social distancing.

Congruent with our pre‐registered hypotheses, our results demonstrated that the best predictor of people’s adherence to distancing was perceived adherence of their close circle, which exceeded the effect of people’s own approval of the rules. Perceived adherence of fellow citizens only mattered for people closely bonded with their country. Across the social scales, perceived adherence of others was a better predictor than perceived approval of others. Moreover, perceived vulnerability of loved ones predicted adherence in addition to perceived self‐vulnerability to the disease, and self‐vulnerability impacted adherence more strongly for people with larger close circles. Extending the growing body of literature on the consequences of the COVID‐19 pandemic, this study uniquely shows the role of social influence in driving people’s adherence to the distancing rules in a global sample.

Building upon the pre‐COVID‐19 literature on social group formation, imitation, and bonding, our findings show how social influence from one’s close circle guides behavioural change during a crisis. We know that social bonds are formed and entrenched via a well‐established mechanism of selective social learning from – and imitation of – bonded others (Chartrand & Lakin, 2011; Haun & Over, 2015). People tend to trust, agree, favour, and cooperate more with those in their close circles (Raafat et al., 2009). Supporting previous research on social norm change (Drury, 2018; Mawson, 2005; Prentice & Paluck, 2020), our results indicate that in the rapidly changing and threatening pandemic situation, people had an increased need to turn towards their bonded inner groups for reference – whether that be their close circle of family and friends or fellow citizens.

Importantly, our study focussed on people’s perceptions of what others did and thought, rather than measuring others’ objective behaviour. Thus, we capture the role of social *expectations* in norm change, demonstrated previously in numerous laboratory and field experiments (Bicchieri, 2016; Borgonovi & Andrieu, 2020). *Expecting* that others follow the new rule is crucial for encouraging people’s adherence. Our data suggest that widespread adherence to pandemic rules can be achieved by highlighting that others in one’s close circle and community are complying to the rules, for instance, by encouraging people within bonded groups to communicate about their good behaviour and encourage others to follow them (Andrews et al., 2020). Notably, this is different from the current approach of warning people about the disease threat or persuading them that distancing is individually or globally the right thing to do through appeals to general pro‐social behaviour, which have been shown to be ineffective (Favero & Pedersen, 2020).

Exploratory analyses showed that beliefs in collective efficacy and collectivism also predicted adherence. Despite considerable variability among the top 10 countries in our data set, three predictors of adherence stood out: perceived adherence of close others, own approval of the rules, and belief in the collective efficacy of actions taken. Previous research with smaller samples has shown how empathy and collectivism can enhance individuals’ intention to engage in social distancing (Pedersen & Favero, 2020). Our study further demonstrates how collective efficacy beliefs and collectivism influence adherence more strongly than selfish motives such as vulnerability to the disease.

Evidencing the impact of close social bonds, perceived vulnerability of both self and others was more strongly associated with adherence for people with a close circle of two or more people. An enhanced sense of threat for loved ones may have motivated those with larger close circles to adhere more. Yet, why did self‐vulnerability, seemingly the most selfish factor we assessed, predicted adherence more for those with a larger close circle? Research on health and self‐care behaviour shows that social support can motivate chronic patients to make sustained healthy lifestyle changes (Gallagher et al., 2011; Heaney & Israel, 2008; Tang et al., 2008). Social support in the face of a threatening, fear‐invoking situation can trigger behavioural change by facilitating one’s belief in their ability to cope (Witte & Allen, 2000). Similarly, our participants with larger close circles may have felt more supported, thus transforming their negative feelings of vulnerability into problem‐solving and adherence to distancing rules (Jetten et al., 2020).

A limitation of this study, shared with most existing empirical studies on COVID‐19, is the difficulty of parsing out causal relationships due to collecting self‐report measures with no pre‐pandemic baseline available. For instance, people’s responses about their close circle’s adherence may be reflecting how well they themselves have been adhering to distancing. Yet, given converging evidence showing the role of social norms on COVID‐19 adherence (Borgonovi & Andrieu, 2020; Lin et al., 2020; Nakayachi et al., 2020), it is more likely that our results demonstrate this link rather than merely reflect the projection of one’s actions onto others. Additionally, there are inherent drawbacks related to sampling bias in online studies. Our sample is comprised largely of educated young women, which incurs some limits on the generalizability of our findings for the general population. Still, our large sample size and the fact that all analyses were adjusted for these demographic variables mean that the findings remain highly informative. Using our open‐access data set and those of many other COVID‐19 studies, future research can provide more specific insights, for instance, on potential gender, cross‐cultural, and socio‐economic differences in people’s responses to the pandemic.

These findings have several key policy implications. Beyond convincing individuals about the threat of the disease or the necessity of adherence to the new rules, the influences of close circles should be given consideration. Firstly, when rapid behavioural change is needed, people’s decisions on whether to adhere to the new rules depend on their perception of others’ adherence. When others within a bonded community follow new rules, everyone is more likely to start adopting them, even if they have not yet fully internalized the value of these rules, which could be a lengthier process. Thus, an effective strategy could be to simply directly ask people to encourage their loved ones and communities to adhere to the measures. Secondly, it should be acknowledged that following what others in one’s close circle do could also lead to a failure to adhere to the new norms, if the close circle displays poor rule‐following. Therefore, ensuring that a sense of community and shared future is created at a large‐scale (i.e., with fellow citizens in the country) alongside the small‐scale is essential. Finally, to promote adherence to pandemic‐related measures, public messages should emphasize collectivistic values (e.g., working for the benefit of the community) and the efficacy of the collective actions. For effective policies during pandemics and future crises that require a collective behavioural response, our message is as follows: Even when the challenge is to practise social distancing, social closeness is the solution.

## Conflicts of interest

All authors declare no conflict of interest.

## Author contributions

Bahar Tuncgenc (Conceptualization; Data curation; Formal analysis; Investigation; Methodology; Project administration; Visualization; Writing – original draft) Marwa El Zein (Conceptualization; Formal analysis; Investigation; Methodology; Writing – original draft) Justin Sulik (Conceptualization; Data curation; Formal analysis; Investigation; Methodology; Software; Visualization; Writing – review & editing) Martha Newson (Investigation; Methodology; Writing – review & editing) Yi Zhao (Data curation; Formal analysis; Writing – review & editing) Guillaume Dezecache (Conceptualization; Investigation; Methodology; Writing – review & editing) Ophelia Deroy (Conceptualization; Funding acquisition; Investigation; Methodology; Writing – review & editing).

## Supporting information

Supplementary Materials

## Data Availability

The raw data and scripts used for the statistical analyses will be available on our study’s OSF page https://osf.io/hyjq9 upon publication of all pre‐registered aims. The OSF repository includes other pre‐registered aims not covered in this paper; sister‐papers are currently in progress for reporting on those aims. Researchers requesting access to the data set before it is made publicly available should contact the first author.
