# Supplementary material for: Social influence matters: We follow pandemic guidelines most when our close circle does
Source: Br J Psychol. 2021 Jan 20;112(3):763–80. doi: 10.1111/bjop.12491 (PMC8014579; doi:10.1111/bjop.12491)
Supplement: Supplementary file 1 — Supplementary Materials [file BJOP-112-763-s001.docx]

Supplemental Materials for:

**Social influence matters: We follow pandemic guidelines most when our close circle does**

**Supplemental Methods**

The survey script was built using jsPsych^55^ (de Leeuw, 2015) and can be accessed on the study’s Open Science Framework (OSF) page. The full survey for the first time-point included several tasks not relevant to the research questions reported in the main text; we will report those results in other articles. We detail these additional tasks below, referring to their position in the survey by names given in the main text. The survey used in time-points 2 to 6 were much briefer and included only the following questions: close circle size (together with questions about modes of interaction and whether the contacts resided in the same country or house as the participant), adherence, approval, vulnerability, mood and wellbeing, going out, and certain demographics (i.e., student/work status, current place of residence and number of people in household).

The survey started with the **close social circle** questions that spanned across three screens. First, participants were asked to write the names or initials of up to 20 people “with whom you voluntarily had a conversation in the PAST 7 DAYS and how you interacted with them. Select all that apply. Please do not include casual contacts, such as compulsory work meetings and interactions with shop workers or professionals (e.g., doctors, police, etc.).”. As options for modes of interaction, participants were asked to select among the options of: Face to face, video chat, phone, email, message, other. On the next screen, participants were provided with the names they have just listed and for each person on the list, asked to indicate whether they would “turn to these people if you wanted to seek advice or comfort if you had a major personal problem”. On the last screen of the close social circle questions, participants were asked to indicate whether each contact was currently living in the same household and/or the same country as themselves.

The next set of questions in the survey were about **social norm change** and **vulnerability**, both of which are described in full detail in the main text.

|  |
| --- |
|  |
| **Figure S1. Two user interfaces from the online survey.**   1. Survey item assessing the size of participants’ close social circle. In a previous step, participants had entered the names of those they had interacted with in the last seven days. On the screen shown here, they classed each name according to whether they would seek advice or comfort from that person or not. The size of participants’ close circle is the number of people whom they would turn to for comfort or advice. 2. Survey item assessing identity fusion with country, following Swann et al. (2009). |

After the vulnerability questions, participants rated their **mood** **and wellbeing** over the previous week using the 7-item Warwick-Edinburgh Mental Well-being Scale^56^ (Tennant et al., 2007). The Persian version of this scale was not available on the scale’s official website and was therefore translated and back-translated by native speakers proficient in English before use. Along with this scale, we included four general mood items, asking participants to what extent they had been feeling depressed, anxious, angry and lonely. All of these items were answered on a 5-point Likert type scale with the response options: 1 = None of the time, 2 = Rarely, 3 = Some of the time, 4 = Often, 5 = All of the time.

The next screen included 5 items all answered on continuous slider scales. There were two items about participants’ belief in **collective responsibility** and two about participants’ belief in the **collective efficacy** of the measures being taken (details in the main text). This was followed by a single-item question asking the participants how much they have been **going out** in the past 7 days, with the response options ranging from 1 = Much less than usual through 50 = About the same as usual to 100 = Much more than usual. This single-item going out question was also repeated in the time-points 2 to 6 of the survey.

After that, participants rated their agreement with 3 items probing their belief in the **credibility of science** (adapted from Hartman et al., 2017), using continuous sliders ranging from “completely disagree” to “completely agree”. The items were:

1. People trust scientists a lot more than they should
2. A lot of scientific theories are dead wrong
3. Our society places too much emphasis on science

The credibility of science scale was followed by two **fusion** questions (see Figure S1B for an example), asking the participants how close or distant they felt (i) to the country they are currently living in and (ii) to the government of that country. Both of these questions had an opt-out option in case participants did not feel comfortable indicating their views about their country on an online platform. On the same screen, after the fusion questions, participants rated their **political ideology** on a continuum from "very liberal" to "very conservative", with two opt-out options: "Does not apply to my political views" and "Prefer not to say".

Following this, on two subsequent screens, the 8-item **vertical collectivism** scale (Singelis et al., 1995) and the 15-item **emotion quotient** scale (Wakabayashi et al., 2006) were presented. Items included in these scales are presented in Tables S1.

| *Table S1. Instructions and items used to assess wellbeing (adopted from Tennant et al., 2007), vertical collectivism (adopted from Singelis et al., 1995) and emotion quotient (adopted from Wakabayashi et al., 2006).* |
| --- |
| **Wellbeing** |
| *Below are some statements about feelings and thoughts.*  *Please tick the box that best describes your experience of each over* *THE LAST WEEK.*  [Answer options: 1 = None of the time, 2 = Rarely, 3 = Some of the time, 4 = Often, 5 = All of the time] |
| 1. I’ve been feeling optimistic about the future |
| 1. I’ve been feeling useful |
| 1. I’ve been feeling relaxed |
| 1. I’ve been dealing with problems well |
| 1. I’ve been thinking clearly |
| 1. I’ve been feeling close to other people |
| 1. I’ve been able to make up my own mind about things |
| **Mood** |
| 1. I’ve been feeling depressed |
| 1. I’ve been feeling anxious |
| 1. I’ve been feeling angry |
| 1. I’ve been feeling lonely |
| **Vertical Collectivism** |
| *Please answer the below questions based on how you think and feel GENERALLY.*  [Answer options are presented on a 9-point Likert scale, where 1 = Never, 9 = Always] |
| 1. I would do what would please my family, even if I detested that activity |
| 1. Before taking a major trip, I consult with most members of my family and many friends |
| 1. I usually sacrifice my self-interest for the benefit of my group |
| 1. Children should be taught to place duty before pleasure |
| 1. I hate to disagree with others in my group |
| 1. We should keep our aging parents with us at home |
| 1. Children should feel honoured if their parents receive a distinguished award |
| **Emotion Quotient** |
| *How strongly do you agree or disagree with the following statements? There are no right or wrong answers, or trick questions.*  [Answer options: 1 = Strongly disagree, 2 = Slightly disagree, 3 = Slightly agree, 4 = Strongly agree] |
| 1. I am good at predicting how someone will feel |
| 1. I am quick to spot when someone in a group is feeling awkward or uncomfortable |
| 1. I can sense if I am intruding, even if the other person doesn't tell me |
| 1. I can tune into how someone else feels rapidly and intuitively |
| 1. I can easily work out what another person might want to talk about |
| 1. I find it difficult to explain to others things that I understand easily, when they don't understand it first time |
| 1. I find it hard to know what to do in a social situation |
| 1. Friendships and relationships are just too difficult, so I tend not to bother with them |
| 1. I often find it difficult to judge if something is rude or polite |
| 1. I don't tend to find social situations confusing |
| 1. I really enjoy caring for other people |
| 1. If I say something that someone else is offended by, I think that that's their problem, not mine |
| 1. Seeing people cry doesn't really upset me |
| 1. I usually stay emotionally detached when watching a film |
| 1. I tend to get emotionally involved with a friend's problems |

Finally, a set of demographics questions were asked. Namely, the survey asked participants to indicate (i) their age, (ii) their gender, (iii) highest education completed, (iv) whether they were currently a student or working (with the sub-questions of full-time vs half-time vs part-time and whether they were studying/working from home), (v) their current country, state/province and city of residence, (vi) the country, state/province and city where they grew up, (vii) number of people in their household (with sub-questions of how many children and people above 60 years old), and (viii) their occupation. The occupation options were adapted from the International Standard Classification of Occupations (International Labour Office, 2012).

**Supplemental Results**

Detailed information about the priors, posterior distributions, and model fit parameters for the Bayesian analyses reported in the main text can be found on the study’s OSF page. We also publish the R script used on the same OSF page *[blinded for peer-review]*.

Table S2 shows the full demographic information about the two datasets used in our analyses reported in the main text.

| *Table S2. Demographics of the two datasets used in our regression models.* | | | |
| --- | --- | --- | --- |
|  | **Dataset 1 (n= 5335)** | **Dataset 2 (n= 6634)** | |
| **Models used** | ‘social adherence’ model, ‘social approval’ model, exploratory model | vulnerability model | |
| **Mean age (SD)** | 37.05 (14.26) | 36.55 (14.23) | |
| **Gender (n)** | Woman: 3703  Man: 1545  Non-binary: 53  None: 34 | Woman: 4333  Man: 2190  Non-binary: 59  None: 52 | |
| **Education (n)** | *No schooling completed:* 7  *Primary education (age: 5-10):* 8  *Secondary education (age: 11-17):* 759  *University undergraduate degree or professional equivalent:* 2388  *Postgraduate degree:* 2173 | *No schooling completed:* 12  *Primary education (age: 5-10):* 17  *Secondary education (age: 11-17):* 1064  *University undergraduate degree or professional equivalent:* 3081  *Postgraduate degree:* 2460 | |
| **Time spent home (n)** | *Yes, most of the time:* 2692  *Sometimes:* 953  *No, rarely:* 847  Other: 843 | *Yes, most of the time:* 3191  *Sometimes:* 1231  *No, rarely:* 1110  Other: 1102 | |
| **Work status** | *Full-time work:* 2675  *Part-time work:* 329  *Half-time work:* 526  *Full-time study:* 1211  *Part-time study:* 383  *Neither*: 211 | *Full-time work:* 3172  *Part-time work:* 657  *Half-time work:* 367  *Full-time study:* 1537  *Part-time study:* 589  *Neither*: 312 | |
| **Top 10 countries with largest sample sizes** | UK: 1829, Turkey: 792, USA: 475,  France: 322, Peru: 320, Germany: 213, Bangladesh: 142, Sweden: 127,  Australia: 126, Italy: 103 | | UK: 1937, Turkey:1148, Peru: 724, USA: 543, France: 344,  Bangladesh: 275, Germany: 221, Sweden: 138, Australia: 135, Italy: 112 |

**Vulnerability Model**

Since this model did not include close social circle size or fusion with the country variables, missing values on these two variables did not affect this model and the results reported in the main text were run with the larger dataset of 6634 participants. To confirm that the findings held when these participants are excluded, we re-ran the same model with the smaller dataset as well. The results showed that all of the found effects were retained (see Table S3).

| *Table S3. Results of the vulnerability model conducted with the small dataset. The outcome variable was self-adherence to distancing behaviour and random effects of country was included. The model was adjusted for participant age, gender, education level (four levels), time spent outside of home (three levels: mostly, sometimes, rarely) and country’s stringency of lockdown measures in the participant’s country of residence.* | | | |
| --- | --- | --- | --- |
|  | **β** | **SE** | **95% Credible Intervals** |
| Vulnerability_self_ | 0.13 | 0.02 | 0.10, 0.16 |
| Vulnerability_others_ | 0.12 | 0.02 | 0.09, 0.15 |
| Close circle size | 0.02 | 0.02 | -0.01, 0.05 |
| Vulnerability_self_ x close circle size | 0.04 | 0.02 | 0.01, 0.07 |
| Vulnerability_others_ x close circle size | 0.00 | 0.02 | -0.03, 0.03 |

**Supplemental References**

de Leeuw, J.R. (2015). jsPsych: A JavaScript library for creating behavioral experiments in a Web browser. *Behavior Research Methods*, 47(1), 1-12. doi:10.3758/s13428-014-0458-y

Hartman, R. O., Dieckmann, N. F., Sprenger, A. M., Stastny, B. J., & DeMarree, K. G. (2017). Modeling attitudes toward science: development and validation of the credibility of science scale. *Basic and Applied Social Psychology*, 39(6), 358-371.

International Labour Office (2012). International Standard Classification of Occupations. Vol. 1, Geneva. Retrieved: <https://www.ilo.org/wcmsp5/groups/public/---dgreports/---dcomm/---publ/documents/publication/wcms_172572.pdf>.

Singelis, T. M., Triandis, H. C., Bhawuk, D. P. S., & Gelfand, M. J. (1995). Horizontal and vertical dimensions of individualism and collectivism: A theoretical and measurement refinement. Cross-Cultural Research, 29(3), 240–275. https://doi.org/10.1177/106939719502900302

Tennant, R., Hiller, L., Fishwick, R., Platt, S., Joseph, S., Weich, S., Parkinson, J., Secker, J. & Stewart-Brown, S. (2007). The Warwick-Edinburgh mental well-being scale (WEMWBS): development and UK validation. *Health and Quality of life Outcomes*, 5(1), 63.

Wakabayashi, A., Baron-Cohen, S., Wheelwright, S., Goldenfeld, N., Delaney, J., Fine, D., … Weil, L. (2006). Development of short forms of the Empathy Quotient (EQ-Short) and the Systemizing Quotient (SQ-Short). Personality and Individual Differences, 41(5), 929–940. https://doi.org/10.1016/j.paid.2006.03.017.
